# Supplementary material for: A Likelihood Approach for Real-Time Calibration of Stochastic Compartmental Epidemic Models
Source: PLoS Comput Biol. 2017 Jan 17;13(1):e1005257. doi: 10.1371/journal.pcbi.1005257 (PMC5240920; doi:10.1371/journal.pcbi.1005257)
Supplement: S1 File — (TAR.GZ) [file pcbi.1005257.s014.tar.gz › HSPH_Online-SI-Revision/MSS10/n10-mild/n10-mild_NEW_table-4.pdf]

1	R0	{1.25046, 6.80278, 0.1}	{1.07118, 2.22306, 0.06}	{{1.06738, 2.19254, 0.}, {1.06738, 2.19254, 0.}}	{14.3368, 67.3213, 152.}	inf	157.	42.7422	{9.8825, 80.949}	72.7757	0.665068	newly	307.	136.22	{32., 252.}	44.2147	0.305353	Reff	1.16118	1.00694	{0.998786, 1.00699}	13.2831	ar	3516.	1401.03	{641., 2191.}	60.1931
2	R0	{1.32013, 4.45847, 0.1}	{1.24392, 3.13642, 0.01}	{{1.22544, 2.5364, 0.}, {1.25474, 3.26445, 0.}}	{5.773, 29.6526, 108.}	inf	184.	126.743	{63.7618, 190.594}	33.4054	0.210669	newly	307.	329.61	{164., 473.}	32.6026	0.148839	Reff	1.21267	1.14975	{1.13011, 1.16684}	5.18871	ar	4420.	3547.08	{2997., 4090.}	19.8226
3	R0	{1.31785, 5.36796, 1.}	{1.18943, 2.7504, 0.}	{{1.06738, 2.19254, 0.}, {1.25474, 3.26445, 0.}}	{9.74449, 48.7627, 100.}	inf	142.	77.5849	{23.5564, 133.34}	47.6665	0.344335	newly	232.	229.95	{72., 409.}	44.2543	0.221786	Reff	1.23206	1.11966	{1.0022, 1.18078}	9.12286	ar	4942.	2871.84	{1147., 3857.}	41.8891
4	R0	{1.38801, 6.16031, 0.1}	{1.19342, 2.85698, 0.}	{{1.06738, 2.19254, 0.}, {1.25474, 3.26445, 0.}}	{14.0195, 53.6227, 100.}	inf	202.	86.0093	{35.4097, 133.616}	57.4211	0.418729	newly	273.	231.92	{109., 364.}	31.2967	0.172876	Reff	1.26267	1.0994	{0.984758, 1.15546}	12.9311	ar	4790.	2908.27	{1300., 3773.}	39.2846
5	R0	{1.27955, 11.57, 1.}	{1.1367, 5.81822, 0.08}	{{1.13227, 5.71232, 0.}, {1.13726, 6.0871, 0.}}	{11.1639, 49.7129, 92.}	inf	185.	74.9808	{54.4304, 94.0049}	59.4698	0.401749	newly	121.	90.98	{65., 115.}	25.438	0.136253	Reff	1.13842	1.02383	{1.01794, 1.02679}	10.0656	ar	3460.	2243.36	{1730., 2739.}	35.163
6	R0	{1.22184, 4.34395, 1.}	{1.23329, 3.26167, 0.05}	{{1.08063, 2.5364, 0.}, {1.28596, 3.98513, 0.}}	{3.51059, 24.9146, 95.}	inf	108.	64.7924	{18.3193, 107.22}	43.8765	0.308948	newly	215.	167.41	{57., 256.}	37.3256	0.228985	Reff	1.17138	1.18763	{1.03874, 1.23768}	3.82159	ar	3524.	3418.59	{1849., 4169.}	16.1382
7	R0	{1.23856, 4.59068, 0.1}	{1.23176, 2.79114, 0.05}	{{1.22544, 2.5364, 0.}, {1.25474, 3.26445, 0.}}	{2.25478, 39.1998, 146.}	inf	160.	113.317	{45.8338, 174.15}	35.0482	0.236855	newly	265.	332.64	{131., 510.}	48.2491	0.207334	Reff	1.13155	1.13101	{1.11752, 1.15556}	2.51324	ar	3855.	3414.62	{2763., 4043.}	14.1411
8	R0	{1.34531, 10.6689, 1.}	{1.2364, 3.26991, 0.1}	{{1.21159, 3.07157, 0.}, {1.28596, 3.98513, 0.}}	{8.09582, 69.351, 90.}	inf	312.	101.68	{41.5884, 148.071}	67.4102	0.550908	newly	198.	249.12	{103., 378.}	49.3838	0.209874	Reff	1.20365	1.13219	{1.11054, 1.17412}	5.93684	ar	4852.	3389.83	{2673., 3963.}	30.1354
9	R0	{1.31775, 5.19226, 0.1}	{1.2533, 3.73813, 0.26}	{{1.06738, 2.19254, 0.}, {1.28596, 4.21286, 1.}}	{5.22544, 28.2567, 320.}	inf	312.	88.3353	{36.5958, 137.845}	35.1186	0.223663	newly	202.	189.83	{83., 295.}	32.6782	0.169814	Reff	1.22551	1.16941	{1.00404, 1.20667}	4.90185	ar	4708.	3521.3	{2103., 4365.}	25.2069
10	R0	{1.25916, 6.89207, 0.1}	{1.06766, 2.2418, 0.09}	{{1.06738, 2.19254, 0.}, {1.06738, 2.19254, 0.}}	{15.2082, 67.4727, 172.}	inf	175.	53.5829	{13.7258, 103.133}	69.3812	0.637163	newly	191.	149.08	{42., 294.}	45.6649	0.313164	Reff	1.10151	0.946955	{0.940384, 0.951771}	14.0315	ar	3823.	1606.88	{1146., 2056.}	57.9681
11	R0	{1.20377, 5.46566, 0.1}	{1.36601, 1.42005, 1.15}	{{1.06836, 1.31573, 1.}, {1.42206, 1.81005, 2.}}	{17.0772, 74.0187, 1052.}	inf	147.	185.104	{24.7756, 426.285}	81.6903	0.388102	newly	200.	1541.19	{110., 3087.}	685.825	0.815981	Reff	1.11577	1.2603	{1.00031, 1.33368}	16.3199	ar	3114.	4506.9	{1511., 5598.}	65.5735
12	R0	{1.27878, 2.55804, 0.1}	{1.34664, 2.16032, 0.02}	{{1.24751, 1.89883, 0.}, {1.39162, 2.5364, 0.}}	{6.92072, 17.9963, 118.}	inf	57.	76.4679	{15.0131, 132.73}	71.8745	0.294762	newly	277.	427.37	{83., 879.}	91.4765	0.32876	Reff	1.24259	1.30585	{1.20906, 1.35808}	6.76694	ar	3930.	4550.02	{3555., 5256.}	21.9542
13	R0	{1.41227, 5.45696, 0.1}	{1.24422, 2.75096, 0.08}	{{1.22544, 2.5364, 0.}, {1.24751, 3.07157, 0.}}	{11.8993, 49.8996, 166.}	inf	188.	112.479	{35.0727, 190.54}	42.7398	0.308785	newly	295.	338.62	{144., 561.}	42.861	0.198404	Reff	1.28587	1.14217	{1.12346, 1.15673}	11.1754	ar	4822.	3448.56	{2685., 4032.}	28.939
14	R0	{1.32917, 7.27593, 1.}	{1.53891, 9.38038, 1.48}	{{1.28596, 5.40945, 0.}, {2.1968, 19.9579, 3.}}	{17.6593, 49.4876, 132.}	inf	250.	365.282	{171.462, 850.634}	65.8426	0.198373	newly	248.	287.99	{224., 347.}	22.4476	0.093542	Reff	1.18482	1.34513	{1.15711, 1.82628}	15.1101	ar	4758.	5476.41	{4198., 8432.}	23.8775
15	R0	{1.31629, 9.97481, 2.}	{1.20805, 3.32756, 0.38}	{{1.06738, 2.5149, 0.}, {1.28596, 3.98513, 1.}}	{8.22348, 66.6404, 81.}	inf	186.	75.9489	{40.4762, 117.474}	59.1673	0.435126	newly	149.	182.8	{105., 304.}	42.0403	0.168898	Reff	1.22178	1.13452	{1.00828, 1.20631}	7.14227	ar	4543.	3088.22	{1637., 3975.}	32.0225
16	R0	{1.36735, 5.74166, 0.1}	{1.23985, 3.14863, 0.07}	{{1.22544, 2.5364, 0.}, {1.28596, 3.98513, 0.}}	{9.39533, 45.1616, 162.}	inf	182.	105.012	{41.5614, 167.787}	43.1937	0.313108	newly	266.	275.65	{111., 413.}	35.8759	0.180716	Reff	1.26507	1.15658	{1.13884, 1.19514}	8.64725	ar	4516.	3549.54	{2428., 4231.}	21.7086
17	R0	{1.27524, 13.2396, 0.1}	{1.84149, 18.8301, 0.98}	{{1.80508, 18.4377, 1.}, {1.86254, 19.1994, 1.}}	{44.4031, 43.7026, 884.}	inf	80.	105.389	{89.796, 123.006}	32.8553	0.121299	newly	45.	44.91	{36., 55.}	13.4	0.0594658	Reff	1.24591	1.7944	{1.76013, 1.81663}	44.0232	ar	3731.	7450.58	{7248., 7646.}	99.6939
18	R0	{1.21884, 7.71545, 1.}	{1.11332, 2.9175, 0.71}	{{1.06738, 2.19254, 0.}, {1.22544, 3.57392, 1.}}	{9.01207, 62.1863, 43.}	inf	167.	54.6339	{15.9655, 91.0637}	67.2851	0.560843	newly	159.	135.49	{47., 215.}	37.3774	0.225128	Reff	1.1234	1.03862	{0.993997, 1.14254}	8.33564	ar	3080.	1907.86	{906., 3274.}	41.6201
19	R0	{1.34879, 6.71464, 0.1}	{1.23537, 2.68749, 0.}	{{1.22544, 2.5364, 0.}, {1.24751, 3.07157, 0.}}	{8.40898, 59.9756, 100.}	inf	339.	122.37	{51.4618, 192.154}	63.9026	0.493321	newly	410.	346.19	{144., 524.}	31.3049	0.18039	Reff	1.15092	1.0809	{1.06555, 1.10037}	6.08361	ar	4585.	3316.16	{2618., 3887.}	27.6737
20	R0	{1.37284, 5.75036, 0.1}	{1.15834, 2.62568, 0.08}	{{1.06738, 2.19254, 0.}, {1.25474, 3.26445, 0.}}	{15.6244, 54.3389, 172.}	inf	216.	94.8583	{37.1611, 155.691}	56.0841	0.425179	newly	303.	260.93	{99., 418.}	35.099	0.202893	Reff	1.23597	1.05678	{0.971822, 1.1432}	14.4981	ar	4750.	2573.56	{1189., 3730.}	45.8198
21	R0	{1.29938, 5.36419, 0.1}	{1.38366, 5.19664, 0.55}	{{1.22544, 2.5364, 0.}, {2.01498, 14.4607, 3.}}	{13.3396, 64.1717, 612.}	inf	198.	219.697	{78.2641, 595.228}	62.9322	0.249751	newly	270.	347.76	{190., 483.}	39.5333	0.146442	Reff	1.18387	1.25242	{1.11963, 1.7543}	11.5954	ar	4337.	4373.63	{3111., 7969.}	29.4183
22	R0	{1.31882, 7.47626, 0.1}	{1.43139, 8.53078, 1.42}	{{1.06738, 2.19254, 0.}, {1.70173, 15.1079, 3.}}	{17.5947, 77.7659, 1418.}	inf	227.	263.494	{47.2931, 481.581}	77.3175	0.371689	newly	235.	245.22	{131., 351.}	24.7149	0.121912	Reff	1.193	1.28515	{0.988622, 1.50335}	15.3981	ar	3977.	4859.04	{2236., 7033.}	45.4156
23	R0	{1.26215, 3.46375, 0.1}	{2.35444, 14.888, 1.37}	{{1.68297, 7.5059, 1.}, {2.86501, 19.6025, 2.}}	{86.635, 331.659, 1286.}	inf	117.	603.447	{302.72, 815.824}	416.989	0.679716	newly	283.	367.19	{311., 433.}	30.1943	0.110946	Reff	1.18314	2.08388	{1.55118, 2.4632}	76.2443	ar	3607.	8367.46	{6820., 9274.}	132.186
24	R0	{1.16712, 4.19464, 1.}	{1.08018, 1.97832, 1.26}	{{1.06738, 1.81005, 0.}, {1.06836, 2.19254, 2.}}	{8.41364, 52.8371, 100.}	inf	122.	68.5352	{17.1218, 117.666}	46.5502	0.375483	newly	227.	227.15	{61., 415.}	47.793	0.283359	Reff	1.04609	0.973896	{0.958035, 0.970088}	7.89761	ar	3000.	1798.47	{1092., 2491.}	42.727
25	R0	{1.22751, 3.34517, 0.1}	{1.67638, 2.75941, 0.02}	{{1.39162, 1.89883, 0.}, {2.13532, 4.08009, 0.}}	{36.5676, 27.9503, 116.}	inf	50.	74.0845	{12.5576, 141.347}	81.7015	0.309573	newly	113.	465.64	{92., 874.}	323.894	0.558834	Reff	1.20861	1.64582	{1.36552, 2.09874}	36.1754	ar	3086.	6414.96	{4865., 8315.}	109.803
26	R0	{1.18747, 5.88083, 1.}	{1.0817, 2.20973, 0.12}	{{1.06738, 2.19254, 0.}, {1.06836, 2.19254, 0.}}	{9.69144, 62.4248, 100.}	inf	178.	76.0931	{32.8546, 123.864}	57.2511	0.417673	newly	234.	215.01	{81., 363.}	36.8248	0.195748	Reff	1.02301	0.942888	{0.925812, 0.936421}	8.78872	ar	3614.	1927.96	{1352., 2530.}	46.7886
27	R0	{1.29023, 4.76202, 0.1}	{1.19464, 2.44127, 0.06}	{{1.06738, 2.19254, 0.}, {1.24751, 2.5364, 0.}}	{7.40873, 48.7347, 154.}	inf	206.	98.6071	{40.7141, 157.084}	52.4834	0.380799	newly	330.	318.21	{132., 526.}	40.1485	0.204714	Reff	1.17217	1.09803	{0.981611, 1.1524}	6.325	ar	4245.	2975.93	{1388., 3970.}	30.0615
28	R0	{1.2258, 12.9056, 3.}	{1.1323, 5.65249, 0.08}	{{1.13227, 5.71232, 0.}, {1.13227, 5.71232, 0.}}	{7.62756, 56.2013, 97.3333}	inf	188.	74.0909	{51.5706, 93.0786}	60.59	0.416403	newly	113.	90.29	{60., 117.}	23.354	0.12454	Reff	1.0369	0.970709	{0.967955, 0.973815}	6.38393	ar	3639.	2240.38	{1799., 2642.}	38.4342
29	R0	{1.34702, 6.29049, 0.1}	{1.19824, 2.98606, 0.08}	{{1.06738, 2.19254, 0.}, {1.25474, 3.57392, 0.}}	{11.0448, 52.5305, 164.}	inf	264.	112.054	{44.5766, 172.071}	57.5551	0.436688	newly	312.	275.77	{117., 407.}	29.5032	0.177495	Reff	1.19265	1.07898	{0.964845, 1.13391}	9.53107	ar	4560.	3047.74	{1713., 3891.}	33.1636
30	R0	{1.37931, 6.78741, 1.}	{1.1308, 2.59137, 0.07}	{{1.06738, 2.19254, 0.}, {1.22544, 3.26445, 0.}}	{18.0172, 61.821, 93.}	inf	251.	87.7207	{27.6068, 161.547}	65.0515	0.546828	newly	287.	224.6	{73., 391.}	38.5157	0.251504	Reff	1.17614	0.98255	{0.924911, 1.07152}	16.4596	ar	4667.	2341.91	{1497., 3423.}	49.8198
31	R0	{1.16062, 4.47529, 0.1}	{2.12278, 11.7406, 1.75}	{{1.44762, 4.00571, 0.}, {2.85956, 19.8999, 3.}}	{82.9003, 171.928, 1730.}	inf	80.	231.806	{65.8779, 382.271}	200.056	0.432585	newly	156.	200.12	{139., 271.}	36.4359	0.134384	Reff	1.12661	2.02162	{1.4024, 2.68668}	79.4423	ar	3037.	7574.3	{5322., 9320.}	149.629
32	R0	{1.37647, 18.7122, 2.}	{1.16745, 5.77332, 0.91}	{{1.17082, 5.77111, 1.}, {1.17082, 5.77111, 1.}}	{15.1849, 69.1468, 54.5}	inf	360.	91.1947	{68.6177, 112.042}	74.6681	0.60309	newly	137.	108.98	{81., 138.}	21.9124	0.113895	Reff	1.09897	0.963475	{0.960448, 0.968769}	12.3295	ar	4824.	2627.54	{2272., 3021.}	45.5319
33	R0	{1.35084, 6.08841, 0.1}	{1.2546, 3.45923, 0.06}	{{1.22544, 3.07157, 0.}, {1.28596, 3.98513, 0.}}	{7.84887, 45.4801, 152.}	inf	217.	123.044	{62.8964, 171.092}	45.0558	0.295211	newly	297.	287.29	{146., 412.}	27.4579	0.139646	Reff	1.23859	1.16193	{1.13072, 1.19039}	6.85961	ar	4759.	3708.05	{2931., 4210.}	23.2782
34	R0	{1.36912, 6.316, 1.}	{1.0918, 2.22767, 0.18}	{{1.06738, 2.19254, 0.}, {1.22544, 2.5364, 0.}}	{20.2553, 64.7298, 100.}	inf	198.	59.8836	{15.4192, 105.686}	69.7558	0.617695	newly	262.	186.05	{43., 311.}	40.5382	0.279224	Reff	1.2459	1.00865	{0.981165, 1.13793}	19.0423	ar	4537.	1785.54	{1022., 2831.}	60.6449
35	R0	{1.35831, 7.09038, 1.}	{1.23168, 2.97288, 0.}	{{1.22544, 2.5364, 0.}, {1.25474, 3.26445, 0.}}	{9.32259, 58.0717, 100.}	inf	331.	150.571	{90.5277, 209.288}	54.5102	0.372842	newly	339.	385.49	{235., 556.}	30.9469	0.131148	Reff	1.17304	1.08585	{1.0732, 1.10848}	7.43215	ar	4667.	3390.46	{2747., 3955.}	27.3525
36	R0	{1.28315, 5.89938, 0.1}	{1.18875, 2.61993, 0.}	{{1.06738, 2.19254, 0.}, {1.24751, 3.07157, 0.}}	{7.35678, 55.5897, 100.}	inf	255.	97.2782	{34.0096, 159.49}	61.8517	0.494516	newly	336.	284.28	{99., 464.}	37.2679	0.230986	Reff	1.15291	1.08671	{0.977883, 1.14573}	5.74203	ar	4460.	2850.18	{1349., 3823.}	36.0946
37	R0	{1.19303, 2.75113, 0.1}	{1.25028, 2.58589, 0.}	{{1.24751, 2.5364, 0.}, {1.24751, 2.5364, 0.}}	{5.2196, 9.47431, 100.}	inf	55.	88.9656	{21.3972, 143.392}	83.6602	0.285086	newly	184.	304.32	{97., 516.}	86.4674	0.28634	Reff	1.13565	1.18586	{1.17331, 1.19242}	4.86272	ar	3142.	3615.43	{3206., 4082.}	19.6967
38	R0	{1.36033, 7.8088, 1.}	{2.14288, 16.6365, 3.}	{{2.14288, 16.6365, 3.}, {2.14288, 16.6365, 3.}}	{57.5265, 113.048, 200.}	inf	249.	709.828	{671.093, 748.335}	185.072	0.454556	newly	256.	349.47	{322., 380.}	36.5117	0.13433	Reff	1.22334	1.82834	{1.8199, 1.83606}	49.4544	ar	4941.	8313.74	{8225., 8403.}	68.2603
39	R0	{1.27353, 3.70845, 0.1}	{1.22044, 2.6906, 0.}	{{1.06738, 2.19254, 0.}, {1.24751, 3.07157, 0.}}	{4.16859, 27.4468, 100.}	inf	114.	87.5592	{24.8075, 150.658}	37.5006	0.244434	newly	257.	263.81	{68., 438.}	43.2257	0.224966	Reff	1.17827	1.13232	{0.997057, 1.16413}	3.8994	ar	3725.	3174.49	{1914., 3812.}	16.1463
40	R0	{1.19983, 12.1486, 0.1}	{1.1414, 5.79241, 0.22}	{{1.13227, 5.71232, 0.}, {1.17082, 6.0871, 1.}}	{4.86999, 52.3204, 276.}	inf	191.	82.3749	{61.3049, 103.595}	56.8718	0.374442	newly	121.	97.7	{70., 123.}	21.8678	0.114557	Reff	1.02525	0.987719	{0.978092, 1.01289}	3.66111	ar	3619.	2305.28	{1781., 2760.}	36.3006
41	R0	{1.20935, 2.67169, 0.1}	{1.21574, 2.84076, 0.04}	{{1.06738, 2.19254, 0.}, {1.25474, 3.26445, 0.}}	{3.89258, 14.3342, 134.}	inf	53.	54.6505	{20.6157, 93.562}	40.4725	0.196353	newly	165.	166.62	{55., 283.}	41.1636	0.205685	Reff	1.16702	1.17296	{1.03281, 1.21127}	3.85527	ar	3429.	3180.68	{1515., 3870.}	17.6127
42	R0	{1.23313, 4.74781, 1.}	{1.18672, 3.10687, 0.29}	{{1.06738, 2.19254, 0.}, {1.28596, 3.98513, 1.}}	{5.8468, 34.5621, 75.}	inf	138.	96.494	{40.3909, 148.146}	34.4375	0.234704	newly	211.	227.8	{115., 336.}	37.2038	0.182395	Reff	1.10217	1.06547	{0.958051, 1.14935}	5.59538	ar	3835.	2820.74	{1347., 3903.}	28.1747
43	R0	{1.26105, 2.39713, 0.1}	{1.25609, 2.5239, 0.03}	{{1.24751, 2.5364, 0.}, {1.24751, 2.5364, 0.}}	{1.646, 6.53569, 126.}	inf	88.	71.284	{21.2306, 120.133}	39.9229	0.279814	newly	321.	265.99	{76., 447.}	42.8505	0.277829	Reff	1.20809	1.20542	{1.19111, 1.20483}	1.46812	ar	3723.	3726.9	{3284., 4068.}	8.99167
44	R0	{1.36948, 8.88006, 0.1}	{1.37886, 6.54043, 1.2}	{{1.21159, 3.07157, 0.}, {1.77553, 15.0866, 4.}}	{15.9192, 65.487, 1228.}	inf	274.	215.603	{50.373, 507.355}	67.5139	0.403779	newly	232.	254.33	{143., 378.}	28.9871	0.124322	Reff	1.23623	1.24839	{1.11326, 1.56356}	13.8102	ar	4437.	4410.26	{2679., 7294.}	36.0712
45	R0	{1.34882, 3.3791, 0.1}	{1.44032, 4.22072, 0.16}	{{1.22544, 3.07157, 0.}, {1.74092, 5.83416, 0.}}	{17.0064, 42.569, 242.}	inf	49.	69.2354	{13.0959, 112.237}	83.3356	0.312941	newly	143.	155.75	{35., 283.}	52.2308	0.26846	Reff	1.32279	1.40727	{1.20012, 1.69689}	16.5691	ar	4422.	4674.85	{3189., 7064.}	31.7447
46	R0	{1.23774, 4.34917, 0.1}	{1.2371, 2.88091, 0.}	{{1.22544, 2.5364, 0.}, {1.25474, 3.26445, 0.}}	{1.7223, 33.7595, 100.}	inf	107.	100.513	{33.8597, 156.196}	33.7134	0.198011	newly	199.	294.4	{107., 503.}	66.6332	0.253867	Reff	1.16533	1.16549	{1.14996, 1.18497}	1.71972	ar	3685.	3487.02	{2704., 4130.}	12.3604
47	R0	{1.34327, 4.78811, 0.1}	{1.81106, 11.2667, 2.15}	{{1.25474, 3.26445, 0.}, {2.01498, 14.4607, 3.}}	{37.55, 147.523, 2094.}	inf	102.	202.97	{76.9234, 284.713}	113.085	0.341759	newly	151.	162.85	{117., 208.}	22.8013	0.0983116	Reff	1.29693	1.72791	{1.21406, 1.91856}	35.8717	ar	4517.	6980.79	{3969., 8097.}	60.4545
48	R0	{1.24366, 4.25814, 0.1}	{1.33183, 2.13678, 0.02}	{{1.24751, 1.89883, 0.}, {1.39162, 2.5364, 0.}}	{7.65481, 49.8189, 118.}	inf	131.	106.304	{17.3564, 184.801}	46.807	0.313612	newly	260.	570.08	{88., 1110.}	145.615	0.388811	Reff	1.16755	1.25343	{1.1736, 1.31885}	7.89452	ar	3678.	4311.05	{3214., 5209.}	27.0253
49	R0	{1.36017, 7.5415, 1.}	{1.06799, 2.25133, 0.12}	{{1.06738, 2.19254, 0.}, {1.06738, 2.19254, 1.}}	{21.4813, 70.1475, 90.}	inf	127.	40.2023	{14.1149, 75.7851}	68.3446	0.574597	newly	116.	118.06	{36., 222.}	53.5	0.26332	Reff	1.22946	0.974624	{0.970026, 0.977161}	20.7273	ar	4178.	1392.69	{920., 1918.}	66.6661
50	R0	{1.25333, 6.63578, 1.}	{1.1438, 2.59167, 0.01}	{{1.06738, 2.19254, 0.}, {1.22544, 3.07157, 0.}}	{8.74798, 60.944, 99.}	inf	201.	86.0825	{41.067, 129.027}	57.1729	0.427281	newly	235.	221.74	{98., 345.}	33.6681	0.182466	Reff	1.0642	0.984263	{0.916505, 1.05987}	7.71557	ar	3401.	2460.44	{1660., 3268.}	28.8433
